# The Russian invasion of Ukraine: a humanitarian tragedy and a tragedy for science

**DOI:** 10.15252/embr.202255164

**Published:** 2022-04-11

**Authors:** Halyna R Shcherbata

**Affiliations:** ^1^ Hannover Medical School Hannover Germany

## Abstract

The Invasion of Ukraine prompts us to support our Ukranian colleagues but also to keep open communication with the Russian scientists who oppose the war.

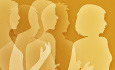

In the eyes of the civilized world, Russia has already lost the war: politically, it is becoming ever more isolated; economically as the sanctions take an enormous toll; militarily as the losses of the Russian army mount. In contrast, the courage of Ukrainian people fighting for their independence has united the Western world that is providing enormous support for those Ukrainians who fight the Russian invasion and those who have fled their war‐torn country. Once this war is over, Ukraine will have to heal the wounds of war, reunite families, restore its economy, reestablish infrastructure, and rebuild science and education. Russia will have to restore its dignity and overcome its self‐inflicted isolation.

Europe’s unity in condemning Russia’s war of aggression and showing its solidarity with Ukraine has been impressive. This includes not the least welcoming and accommodating millions of refugees. We, the scientific community in Europe, have a moral obligation to help Ukrainian students and colleagues by providing safe space to study and to continue their research. First, European research organizations and funding agencies should develop strategies to support them in the years to come. Second, efforts by EMBO, research funders, universities, and research institutions to support Ukrainian students and scientists are necessary. As a first priority, dedicated and unbureaucratic short‐term scholarship and grant programs are required to accommodate Ukrainian scientists; such programs have been already initiated by many organizations, for example, by EMBO, Volkswagen Stiftung, Max Planck Society, and the ERC among others. These help Ukrainian scientists to stay connected to research and become integrated into the European research landscape. In the long‐term and after the war, this aid should be complemented by funding for research centers of excellence in Ukraine, to which scientists could then return.

Even though the priority must be to help Ukrainians, we must also think of students and colleagues in Russia who oppose the war and are affected by the sanctions. As the Iron Curtain closes again, we have to think differently about our ongoing and future collaborations. Although freezing most, if not all, research collaborations with official Russian organizations is justified, it would be a mistake to extend these sanctions to all scientists and students. There is already an exodus of Russian and Belarusian scholars, which will only accelerate in the next months and years, and accepting scientists who ask for political asylum will be beneficial for Europe.

The fraction of Russian society in open opposition to the war is, unfortunately, smaller than that officially in support of it. At the beginning of the war, a number of Russian scientists published an open letter on the internet, in which they condemn this war (https://t‐invariant.org/2022/02/we‐are‐against‐war/). They clearly state that "The responsibility for unleashing a new war in Europe lies entirely with Russia. There is no rational justification for this war”, and “demand an immediate halt to all military operations directed against Ukraine". At the same time, other prominent Russian science and education officials signed the “Statement of the Russian Union of University Rectors (Provosts)”, which expressed unwavering support for Russia, its president and its Army and their goal to “to achieve demilitarization and denazification of Ukraine and thus to defend ourselves from the ever‐growing military threat” (https://www.rsr‐online.ru/news/2022‐god/obrashchenie‐rossiyskogo‐soyuza‐rektorov1/).

Inevitably, Russian scientists must decide themselves how to live and continue their scientific work under the increasingly tight surveillance of the Kremlin regime. History is repeating itself. Not long ago, during the Cold War, Soviet scientists were largely isolated from the international research community and worked in government‐controlled research. In some fields, no one knew what they were working on or where. However, even in those dark times, courageous individuals such as Andrei Sakharov spoke out against the regime and tried to educate the next generation about the importance of free will. Many Soviet geneticists had been arrested under Stalin’s regime of terror and as a result of Lysenkoism and were executed or sent to the Gulag or had to emigrate, such as Nikolaj Timofeev‐Resovskij, one of the great geneticists of his time and an opponent of communism. As a result of sending dissident scientists to Siberia, great educational institutions were created in the region, which trained many famous scientists. History tells us that it is impossible to kill free will and the search for truth.

The Russian invasion of Ukraine is a major humanitarian tragedy and a tragedy for science at many levels. Our hope is that the European science community, policymakers, and funders will be prepared to continue and expand support for our colleagues from Ukraine and eventually help to rebuild the bridges with Russian science that have been torn down.

This commentary has been endorsed and signed by the EMBO Young Investigators and former Young Investigators listed below.

**All signatories are current and former EMBO Young Investigators and endorse the statements in this article.**

**Table:** 

Igor Adameyko	Karolinska Institut, Stockholm, Sweden
Bungo Akiyoshi	University of Oxford, United Kingdom
Leila Akkari	Netherlands Cancer Institute, Amsterdam, Netherlands
Panagiotis Alexiou	Masaryk University, Brno, Czech Republic
Hilary Ashe	Faculty of Life Sciences, University of Manchester, United Kingdom
Michalis Averof	Institut de Génomique Fonctionnelle de Lyon (IGFL), France
Katarzyna Bandyra	University of Warsaw, Poland
Cyril Barinka	Institute of Biotechnology AS CR, Prague, Czech Republic
Frédéric Berger	Gregor Mendel Institute of Molecular Plant Biology, Austrian Academy of Sciences, Vienna, Austria
Vitezslav Bryja	Institute of Experimental Biology, Masaryk University, Brno, Czech Republic
Janusz Bujnicki	International Institute of Molecular and Cell Biology, Warsaw, Poland
Björn Burmann	University Gothenburg, Sweden
Andrew Carter	MRC Laboratory of Molecular Biology, Cambridge, United Kingdom
Pedro Carvalho	Sir William Dunn School of Pathology University of Oxford, United Kingdom
Ayse Koca Caydasi	Koç University, Istanbul, Turkey
Hsu‐Wen Chao	Medical University, Taipei, Taiwan
Jeffrey Chao	Friedrich Miescher Institute, Basel, Switzerland
Alan Cheung	University of Bristol, United Kingdom
Tim Clausen	Research Institute for Molecular Pathology (IMP), Vienna, Austria
Maria Luisa Cochella	The Johns Hopkins University School of Medicine, USA
Francisco Cubillos	Santiago de Chile, University, Chile
Uri Ben‐David	Tel Aviv University, Tel Aviv, Israel
Sebastian Deindl	Uppsala University, Sweden
Pierre‐Marc Delaux	Laboratoire de Recherche en Sciences Végétales, Castanet‐Tolosan, France
Christophe Dessimoz	University, Lausanne, Switzerland
Maria Dominguez	Institute of Neuroscience, CSIC ‐ University Miguel Hernandez, Alicante, Spain
Anne Donaldson	Institute of Medical Sciences, University of Aberdeen, United Kingdom
Peter Draber	BIOCEV, First Faculty of Medicine, Charles University, Vestec, Czech Republic
Xiaoqi Feng	John Innes Centre, Norwich, United Kingdom
Luisa Figueiredo	Institute of Molecular Medicine, Lisbon, Portugal
Reto Gassmann	Institute for Molecular and Cell Biology, Porto, Portugal
Kinga Kamieniarz‐Gdula	Adam Mickiewicz University in Poznań, Poland
Roger Geiger	Institute for Research in Biomedicine, Bellinzona, Switzerland
Niko Geldner	University of Lausanne, Switzerland
Holger Gerhardt	Max Delbrück Center for Molecular Medicine, Berlin, Germany
Daniel Wolfram Gerlich	Institute of Molecular Biotechnology (IMBA), Vienna, Austria
Jesus Gil	MRC Clinical Sciences Centre, Imperial College London, United Kingdom
Sebastian Glatt	Malopolska Centre of Biotechnology, Jagiellonian University, Krakow, Poland
Edgar Gomes	Institute of Molecular Medicine, Lisbon, Portugal
Pierre Gönczy	Swiss Institute for Experimental Cancer Research (ISREC), École Polytechnique Fédérale de Lausanne (EPFL), Lausanne, Switzerland
Maria Gorna	University of Warsaw, Poland
Mina Gouti	Max‐Delbrück‐Centrum, Berlin, Germany
Jerome Gros	Institut Pasteur, Paris, France
Anja Groth	Biotech Research and Innovation Centre (BRIC), University of Copenhagen, Denmark
Annika Guse	Centre for Organismal Studies, Heidelberg, Germany
Ricardo Henriques	Instituto Gulbenkian de Ciência, Oeiras, Portugal
Eva Hoffmann	Center for Chromosome Stability, University of Copenhagen, Denmark
Thorsten Hoppe	CECAD at the Institute for Genetics, University of Cologne, Germany
Yen‐Ping Hsueh	Academia Sinica, Taipei, Taiwan
Pablo Huertas	Andalusian Molecular Biology and Regenerative Medicine Centre (CABIMER), Seville, Spain
Matteo Iannacone	IRCCS San Raffaele Scientific Institute, Milan, Italy
Alvaro Rada‐Iglesias	Institue of Biomedicine and Biotechnology of Cantabria (IBBTEC) University of Cantabria, Santander, Spain
Axel Innis	Institut Européen de Chimie et Biologie (IECB), Pessac, France
Nicola Iovino	MPI für Immunbiologie und Epigenetik, Freiburg, Germany
Carsten Janke	Institut Curie, France
Ralf Jansen	Interfaculty Institute for Biochemistry, Eberhard‐Karls‐University Tübingen, Germany
Sebastian Jessberger	HiFo / Brain Research Institute, University of Zurich, Switzerland
Martin Jinek	University of Zurich, Switzerland
Simon Bekker‐Jensen	University, Copenhagen, Denmark
Nicole Joller	University of Zurich, Switzerland
Luca Jovine	Department of Biosciences and Nutrition & Center for Biosciences, Karolinska Institutet, Stockholm, Sweden
Jan Philipp Junker	Max‐Delbrück‐Centrum, Berlin, Germany
Anna Karnkowska	University, Warsaw, Poland
Zuzana Keckesova	Institute of Organic Chemistry and Biochemistry AS CR, Prague, Czech Republic
René Ketting	Institute of Molecular Biology (IMB), Mainz, Germany
Bruno Klaholz	Institute of Genetics and Molecular and Cellular Biology (IGBMC), University of Strasbourg, Illkirch, France
Jürgen Knoblich	Institute of Molecular Biotechnology (IMBA), Vienna, Austria
Taco Kooij	Centre for Molecular Life Sciences, Nijmegen, Netherlands
Romain Koszul	Institut Pasteur, Paris, France
Claudine Kraft	Institute for Biochemistry and Molecular Biology, Universität Freiburg, Germany
Alena Krejci	Faculty of Science, University of South Bohemia, Ceske Budejovice, Czech Republic
Lumir Krejci	National Centre for Biomolecular Research (NCBR), Masaryk University, Brno, Czech Republic
Arnold Kristjuhan	Institute of Molecular and Cell Biology, University of Tartu, Estonia
Yogesh Kulathu	MRC Protein Phosphorylation & Ubiquitylation Unit, University of Dundee, United Kingdom
Edmund Kunji	MRC Mitochondrial Biology Unit, Cambridge, United Kingdom
Karim Labib	MRC Protein Phosphorylation and Ubiquitylation Unit, University of Dundee, United Kingdom
Thomas Lecuit	Developmental Biology Institute of Marseilles ‐ Luminy (IBDML), France
Gaëlle Legube	Center for Integrative Biology in Toulouse, Paul Sabatier University, France
Suewei Lin	Academia Sinica, Taipei, Taiwan
Ming‐Jung Liu	Academia Sinica, Taipei, Taiwan
Malcolm Logan	Randall Division of Cell and Molecular Biophysics, King’s College London, United Kingdom
Massimo Lopes	University of Zurich, Switzerland
Jan Löwe	Structural Studies Division, MRC Laboratory of Molecular Biology, Cambridge, United Kingdom
Martijn Luijsterburg	University Medical Centre, Leiden, Netherlands
Taija Makinen	Uppsala University, Sweden
Sandrine Etienne‐Manneville	Institut Pasteur, Paris, France
Miguel Manzanares	Spanish National Center for Cardiovascular Research (CNIC), Madrid, Spain
Jean‐Christophe Marine	Center for Biology of Disease, Laboratory for Molecular Cancer Biology, VIB & KU Leuven, Belgium
Sascha Martens	Max F. Perutz Laboratories, University of Vienna, Austria
Elvira Mass	Universität Bonn, Germany
Olivier Mathieu	Clermont Université, Aubière, France
Ivan Matic	Max Planck Institute for Biology of Ageing, Cologne, Germany
Joao Matos	Max Perutz Laboratories, Vienna, Austria
Nicholas McGranahan	University College London, United Kingdom
Hind Medyouf	Georg‐Speyer‐Haus, Frankfurt, Germany
Patrick Meraldi	University of Geneva, Switzerland
Marco Milán	ICREA & Institute for Research in Biomedicine (IRB), Barcelona, Spain
Eric Miska	Wellcome Trust/Cancer Research UK Gurdon Institute, University of Cambridge, United Kingdom
Nuria Montserrat	Institut de Bioenginyeria de Catalunya (IBEC), Barcelona, Spain
Nuno Barbosa‐Morais	Institute of Molecular Medicine, Lisbon, Portugal
Antonin Morillon	Institut Curie, Paris, France
Rafal Mostowy	Jagiellonian University, Krakow, Poland
Patrick Müller	University of Konstanz, Konstanz, Germany
Miratul Muqit	University of Dundee, United Kigdom
Poul Nissen	Centre for Structural Biology, Aarhus University, Denmark
Ellen Nollen	European Research Institute for the Biology of Ageing, University of Groningen, Netherlands
Marcin Nowotny	International Institute of Molecular and Cell Biology, Warsaw, Poland
John O'Neill	MRC Laboratory of Molecular Biology, Cambridge, United Kigdom
Tamer Önder	Koc University School of Medicine, Istanbul, Turkey
Elin Org	University of Tartu, Estonia
Nurhan Özlü	Koç University, Istanbul, Turkey
Bjørn Panyella Pedersen	Aarhus University, Denmark
Vladimir Pena	London, The Institute of Cancer Research, United Kingdom
Camilo Perez	Biozentrum, University of Basel, Switzerland
Antoine Peters	Friedrich Miescher Institute for Biomedical Research (FMI), Basel, Switzerland
Clemens Plaschka	IMP, Vienna, Austria
Pavel Plevka	CEITEC, Masaryk University, Brno, Czech Republic
Hendrik Poeck	Technische Universität, München, , Germany
Sophie Polo	Université Diderot (Paris 7), Paris, France
Simona Polo	IFOM ‐ The FIRC Institute of Molecular Oncology, Milan, Italy
Magdalini Polymenidou	University of Zurich, Switzerland
Freddy Radtke	Swiss Institute for Experimental Cancer Research (ISREC), École Polytechnique Fédérale de Lausanne (EPFL), Lausanne, Switzerland
Markus Ralser	Institute of Biochemistry Charité, Berlin, Germany & MRC National Institute for Medical Research, London, United Kingdom
Jan Rehwinkel	John Radcliffe Hospital, Oxford, United Kingdom
Maria Rescigno	European Institute of Oncology (IEO), Milan, Italy
Katerina Rohlenova	Prague, Institute of Biotechnology, Czech Republic
Guadalupe Sabio	Centro Nacional de Investigaciones Cardiovasculares (CNIC), Madrid, Spain
Ana Jesus Garcia Saez	University of Cologne, CECAD Research Center, Germany
Iris Salecker	Institut de Biologie de l'Ecole Normale Supérieure (IBENS), Paris, France
Peter Sarkies	University of Oxford, United Kingdom
Frédéric Saudou	Grenoble Institute of Neuroscience, France
Timothy Saunders	Centre for Mechanochemical Cell Biology, Interdisciplinary Biomedical Research Building, Warwick Medical School, Coventry, United Kingdom
Orlando D. Schärer	IBS Center for Genomic Integrity, Ulsan, South Korea
Arp Schnittger	Biozentrum Klein Flottbek, University of Hamburg, Germnay
Frank Schnorrer	Aix Marseille University, CNRS, IBDM, Turing Centre for Living Systems, Marseille, France
Maya Schuldiner	Department of Molecular Genetics, Weizmann Institute of Science, Rehovot, Israel
Schraga Schwartz	Weizmann Institute of Science, Rehovot, Israel
Martin Schwarzer	Institute of Microbiology, Academy of Sciences of the Czech Republic
Claus Maria	Instituto de Medicina Molecular Faculdade de Medicina da Universidade de Lisboa, Portugal
Hayley Sharpe	The Babraham Institute, United Kingdom
Halyna Shcherbata	Institute of Cell Biochemistry, Hannover Medical School, Hannover, Germany
Eric So	Department of Haematological Medicine, King's College London, United Kingdom
Victor Sourjik	Max Planck Institute for Terrestrial Microbiology, Marburg, Germany
Anne Spang	Biozentrum, University of Basel, Switzerland
Irina Stancheva	Institute of Cell Biology, University of Edinburgh, United Kingdom
Bas van Steensel	Department of Gene Regulation, The Netherlands Cancer Institute, Amsterdam, Netherlands
Richard Stefl	CEITEC, Masaryk University, Brno, Czech Republic
Yonatan Stelzer	Weizmann Institute of Science, Rehovot, Israel
Julian Stingele	Ludwig‐Maximilians‐Universität, München, Germany
Katja Sträßer	Institute for Biochemistry, University of Giessen, Germany
Kvido Strisovsky	Institute of Organic Chemistry and Biochemistry ASCR, Prague, Czech Republic
Joanna Sulkowska	University, Warsaw, Poland
Grzegorz Sumara	Nencki Institute of Experimental Biology, Warsaw, Poland
Karolina Szczepanowska	International Institute Molecular Mechanisms & Machines PAS, Warsaw, Poland
Luca Tamagnone	Institute for Cancer Research and Treatment, University of Torino Medical School, Italy
Meng How Tan	Singapore, Nanyang Technological University, Singapore
Nicolas Tapon	Cancer Research UK London Research Institute, United Kingdom
Nicholas M. I. Taylor	University, Copenhagen, Denmark
Sven Van Teeffelen	Université de Montréal, Canada
Maria Teresa Teixeira	Laboratory of Molecular and Cellular Biology of Eukaryotes, IBPC, Paris, France
Aurelio Teleman	German Cancer Research Center (DKFZ), Heidelberg, Germany
Pascal Therond	Institute Valrose Biology, University of Nice‐Sophia Antipolis, France
Pavel Tolar	University College London, United Kingdom
Isheng Jason Tsai	Academia Sinica, Taipei, Taiwan
Helle Ulrich	Institute of Molecular Biology (IMB), Mainz, Germany
Stepanka Vanacova	Central European Institute of Technology, Masaryk University, Brno, Czech Republic
Henrique Veiga‐Fernandes	Champalimaud Center for the Unknown, Lisboa, Portugal
Marc Veldhoen	Instituto de Medicina Molecular, Lisbon, Portugal
Louis Vermeulen	Academic Medical Centre, Amsterdam, Netherlands
Uwe Vinkemeier	University of Nottingham Medical School, United Kingdom
Helen Walden	MRC Protein Phosphorylation & Ubiquitylation Unit, University of Dundee, United Kingdom
Michal Wandel	Institute of Biochemistry and Biophysics, PAS, Warsaw, Poland
Julie Welburn	Wellcome Trust Centre, Edinburgh, United Kingdom
Ervin Welker	Institute of Biochemistry, Biological Research Center of the Hungarian Academy of Sciences, Szeged, Hungary
Gerhard Wingender	Izmir Biomedicine and Genome Center, Dokuz Eylul University, Izmir, Turkey
Thomas Wollert	Institute Pasteur, Membrane Biochemistry and Transport, Centre François Jacob, Paris, France
Hyun Youk	University of Massachusetts Medical School, USA
Christoph Zechner	MPI für molekulare Zellbiologie und Genetik, Dresden, Germany
Philip Zegerman	Wellcome Trust / Cancer Research UK Gurdon Institute, University of Cambridge, United Kingdom
Alena Ziková	Institute of Parasitology, Biology Centre AS CR, Ceske Budejovice, Czech Republic
Piotr Ziolkowski	Adam Mickiewicz University, Poznan, Poland
David Zwicker	MPI für Dynamik und Selbstorganisation, Göttingen, Germany

